# Concomitant Morphological Modifications of the Avian Eggshell, Eggshell Membranes and the Chorioallantoic Membrane During Embryonic Development

**DOI:** 10.3389/fphys.2022.838013

**Published:** 2022-04-27

**Authors:** Maeva Halgrain, Sonia Georgeault, Nelly Bernardet, Maxwell T. Hincke, Sophie Réhault-Godbert

**Affiliations:** ^1^ INRAE, Université de Tours, BOA, Nouzilly, France; ^2^ Plate-Forme IBiSA des Microscopies, PPF ASB, Université de Tours and CHRU de Tours, Tours, France; ^3^ Department of Cellular and Molecular Medicine, Faculty of Medicine, University of Ottawa, Ottawa, ON, Canada; ^4^ Department of Innovation in Medical Education, Faculty of Medicine, University of Ottawa, Ottawa, ON, Canada

**Keywords:** chicken, eggshell, chorioallantoic membrane, chorionic epithelium, ultrastructure, demineralization, incubation

## Abstract

The chicken eggshell (ES) consists of 95% calcium carbonate and 3.5% organic matter, and represents the first physical barrier to protect the developing embryo, while preventing water loss. During the second half of development, calcium ions from the inner ES are progressively solubilized to support mineralization of the embryonic skeleton. This process is mediated by the chorioallantoic membrane (CAM), which is an extraembryonic structure that adheres to the eggshell membranes (ESM) lining the inner ES. The CAM surrounds the embryo and all egg contents by day 11 of incubation (Embryonic Incubation Day 11, EID11) and is fully differentiated and functionally active by day 15 of incubation (Embryonic Incubation Day 15, EID15). In this study, we explored the simultaneous morphological modifications in the ES, ESM and the CAM at EID11 and EID15 by scanning electron microscopy. We observed that the tips of the mammillary knobs of the ES remain tightly attached to the ESM fibers, while their bases become progressively eroded and then detached from the bulk ES. Concomitantly, the CAM undergoes major structural changes that include the progressive differentiation of villous cells whose villi extend to reach the ESM and the ES. These structural data are discussed with respect to the importance of ES decalcification in providing the calcium necessary for mineralization of embryo’s skeleton. In parallel, eggshell decalcification and weakening during incubation is likely to impair the ability of the ES to protect the embryo. It is assumed that the CAM could counteract this apparent weakening as an additional layer of physical, cellular and molecular barriers against environmental pressures, including pathogens, dehydration and shocks. However, such hypothesis needs to be further investigated.

## Introduction

The avian eggshell (ES) is a remarkable mineralized structure that isolates and protects the developing embryo from the external environment (Chien Y.-C. et al., 2009). The mineralized calcitic ES is a highly ordered structure which forms upon a bilayered proteinaceous fibrous meshwork, the eggshell membranes (ESM) that are composed of three layers: the outer ESM, the inner ESM, and the limiting membrane (Tan et al., 1992). The ES is the first level of egg defense and acts as a physical barrier against physical trauma, invasion of eggs by microorganisms or exposure to environmental toxins and dehydration (Chien Y.-C. et al., 2009; Maurer et al., 2011). Pores that traverse the eggshell permit the diffusion of metabolic gases (carbon dioxide and oxygen) and water vapor. The dynamics of resorption of the ES is a critical process in embryonic development with considerable biological and economical significance (Maurer et al., 2011). Indeed, ES integrity and ES quality are intimately correlated with the survival and health of chicks and thus are prominent factors in the production of hatchlings (Roberts, 2004).

During the second half of development, the avian embryo uses minerals from egg compartments for mineralization of its skeleton. ES is the major source of Ca^2+^ (about 2.21 g per eggshell) and Mg^2+^ (about 20 mg per eggshell) (Romanoff and Romanoff, 1949) for the developing embryo. Calcium mobilization from the ES during the second half of incubation is concomitant with the progressive mineralization of the embryo skeleton. Notably, the embryo skeleton accounts for more than 80% of the total calcium content of the whole embryo between 11 and 15 days of incubation, and 75% at hatch (Romanoff, 1967). The solubilization of these elements from the inner ES starts around the 11th embryonic incubation day (EID11) (Johnston and Comar, 1955; Romanoff, 1960; Crooks and Simkiss 1974; Freeman and Vince, 1974; Chien Y.-C. et al., 2009; Yair and Uni, 2011), concomitantly with the development and differentiation of the chorioallantoic membrane (CAM). By the end of incubation (EID21), the thinning of the ES resulting from mineral solubilization facilitates the emergence of the hatchling from the egg (Chien et al., 2009a, b).

The CAM is a highly vascularized structure that lines the ESM and develops from EID5 onwards (Romanoff, 1960; Freeman and Vince, 1974). The CAM covers the entire surface of the inner shell membranes by EID11. Between EID8 and EID12, the density of the vascular network increases rapidly to reach its mature morphological structure and growth around EID15 (Ribatti et al., 2006).

It grows rapidly from EID11 to EID15-16, and starts to degrade after day 19 (Romanoff, 1960; Leeson and Leeson, 1963; Narbaitz and Tellier, 1974; Makanya et al., 2016). The CAM is fully differentiated at EID15-16 and is composed of three distinct layers, namely the chorionic (ectodermal) epithelium, the mesoderm and the allantoic (endodermal) epithelium (Leeson and Leeson, 1963; Makanya et al., 2016). They all play different but complementary roles (Gabrielli and Accili, 2010).

Besides mineral metabolism, the CAM is involved in several other vital functions for the embryo and is assumed to play a major role in innate immunity. The CAM chorionic epithelium is in direct contact with the ESM. Thus, the CAM is the first cellular structure to encounter pathogens that penetrate the ES, *via* respiratory pores or microcracks. Its strategic position and its well-developed vasculature allow the recruitment of immune cells locally to the site of bacterial stimulation (Valdes et al., 2002; Hincke et al., 2019). In addition to this cellular response, the CAM has been reported to express antimicrobial molecules, including interferons, as well as other components of innate immunity (Barjesteh et al., 2015; Sharma et al., 2020).

There are several papers showing the structural changes associated with the ES and the ESM (using scanning electron microcopy, SEM) or the chorioallantoic membrane (using histology and transmission electron microscopy) during incubation (Gabrielli and Accili, 2010, Chien et al., 2009a, b; Makanya et al., 2016). However, the modifications occurring at the interface of all these structures simultaneously have not yet been described. In this study, we explored the concomitant morphological modifications of the ES, ESM and the CAM by SEM, at two stages of embryonic development: at EID11 (immature CAM) and EID15 (fully differentiated CAM). The structural observations highlighted in this paper contribute to a better understanding on how some specialized cells of the chorionic epithelium triggers ES demineralization. It also opens new research studies to further investigate the role of the CAM in maintaining egg defense and embryo protection during ES weakening.

## Materials and Methods

### Incubation Procedures and Eggshell, Eggshell Membranes and Chorioallantoic Membrane Sampling

Fertilized eggs were obtained from 33-week broiler hens (Ross 308, Boyé Accouvage, Boissière-en-Gâtine, France) and maintained in the Poultry Experimental Facility UE1295 (INRAE, F-37380 Nouzilly, France, doi: 10.15454/1.5572326250887292E12). Eggs were incubated under standard conditions (45% RH, 37.8°C, automatic turning every hour, Bekoto B64-S, Pont-Saint-Martin, France), after a 3-day storage at 16°C, 85% RH to favor synchronization of developmental stage. For each embryonic day studied (EID11 and EID15), 50 eggs (59.6 ± 2.5 g) containing viable embryos were selected and five eggs were used for analysis by SEM. For egg weight and ES quality (ES weight, breaking strength and thickness), 30 eggs per developmental stages (EID0, non-incubated eggs; EID11; EID15) were analyzed. Egg weight, breaking strength and thickness were determined using Digital Egg Tester 6,000 (Nabel, Kyoto, Japan). ES weight was obtained after ES drying (heating for 2 h at 110°C). Note that the values obtained for the ES weight and thickness include ESM.

At each stage, eggs were opened at the air chamber end and the egg contents were poured into a Petri dish. Embryos were quickly sacrificed by decapitation, in compliance with European legislation on the “Protection of Animals Used for Experimental and Other Scientific Purposes” (2010/63/UE) and guidelines approved by the institutional animal care and use committee (IACUC). For microscopic analysis, five eggs were analyzed at each stage of development. A piece of ES containing ESM and the CAM were removed at the equatorial region of the egg and washed with sterile saline solution (NaCl 0.9%). The three structures (ES, ESM, and CAM) were carefully separated from each other with tweezers. The flattened ESM (inner and outer surfaces) and CAM (inner and outer surfaces) were placed between two pieces of filter paper (Whatman 3 mm CHR, Dominique Dutscher SAS, Bernolsheim, France) in which eyelets (∅0.6 mm) had been punched, such that each surfaces could be visualized by SEM. ES fragments, and ESM and CAM assemblies, were placed in the wells of a 24-well plate containing fixative (4% paraformaldehyde, 1% glutaraldehyde in 0.1 M phosphate buffer, pH 7.2) and stored at 4°C until microscopic analysis.

### Analysis of Eggshell, Eggshell Membranes and Chorioallantoic Membrane by Microscopy

For semi-thin sections, after fixation (3.1), the entire CAM was washed in phosphate buffer and post-fixed by incubation with 2% osmium tetroxide for 1 h. Structures were then fully dehydrated in a graded series of ethanol solutions and embedded in Epon resin, which was allowed to polymerize from 37 to 60°C. Semi-thin sections of these blocks were cut (600 nm) using an ultramicrotome (Leica EM UC7) and stained with toluidine blue (0.5% toluidine blue [Sigma-Aldrich, Missouri, United States) and 1% di-sodium tetraborate (Merck, Darmstadt, Germany)]. Observations were then acquired using an inverted and automated epifluorescence microscope Nikon eclipse Ti2E at ×20 magnification (Nikon Corporation, Minato-ku, Tokyo, Japan) coupled with the software NIS Elements (version 5.30.01, Nikon Corporation, Minato-ku, Tokyo, Japan).

**FIGURE 1:**
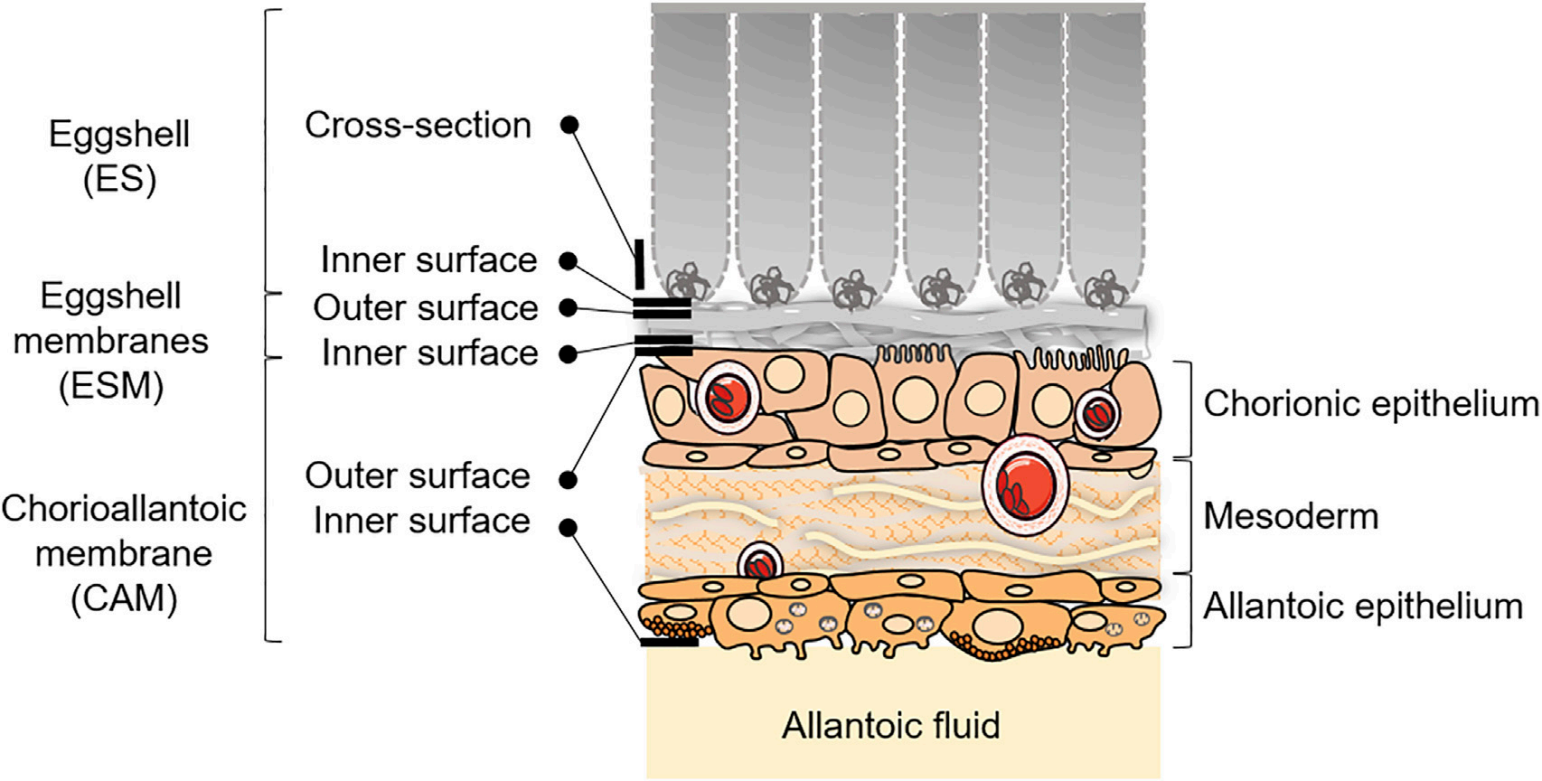
Schematic representation of the ES and CAM, with sampling/visualization strategies depicted for the microscopy analyses at EID11 and EID15. Adapted from Hincke et al., 2019 and Halgrain et al., 2022. Some elements were obtained from Servier Medical Art (https://smart.servier.com), licensed under a Creative Commons Attribution 3.0 Unported License.

For SEM, at each stage, the analysis was performed on the cross section and inner surface of the ES (in contact with the outer side of the ESM), on the outer and inner surfaces of ESM (in contact with the ES or the CAM, respectively) and on the outer and inner surfaces of the CAM (in contact with ESM or with the allantoic fluid). Sampling is illustrated in Figure 1. After fixation (3.1), samples were washed in phosphate buffer and post-fixed by incubation with 2% osmium tetroxide for 1 h. Structures were then fully dehydrated in a graded series of ethanol solutions and dried in hexamethyldisilazane (HMDS, Sigma-Aldrich, Missouri, United States). They were coated with 4 nm carbon, using a GATAN PECS 682 apparatus (Gatan, Pleasanton, United States), prior to analyses using a Zeiss Ultra plus FEG-SEM scanning-electron microscope (Carl Zeiss Microscopy GmbH, Jena, Germany).

### Statistical Analysis

All statistical analysis were performed using R software, version 4.1.1 (R Core Team, 2021, Vienna, Austria). For all egg quality parameters, the normality of the samples was assessed using a Shapiro test and the homogeneity of variance was tested using a Bartlett test. Statistical analyses were performed using ANOVA and post-hoc Tukey HSD tests (*p*-value <0.05).

## Results

### Egg Weight and Eggshell Physical Characteristics

**FIGURE 2:**
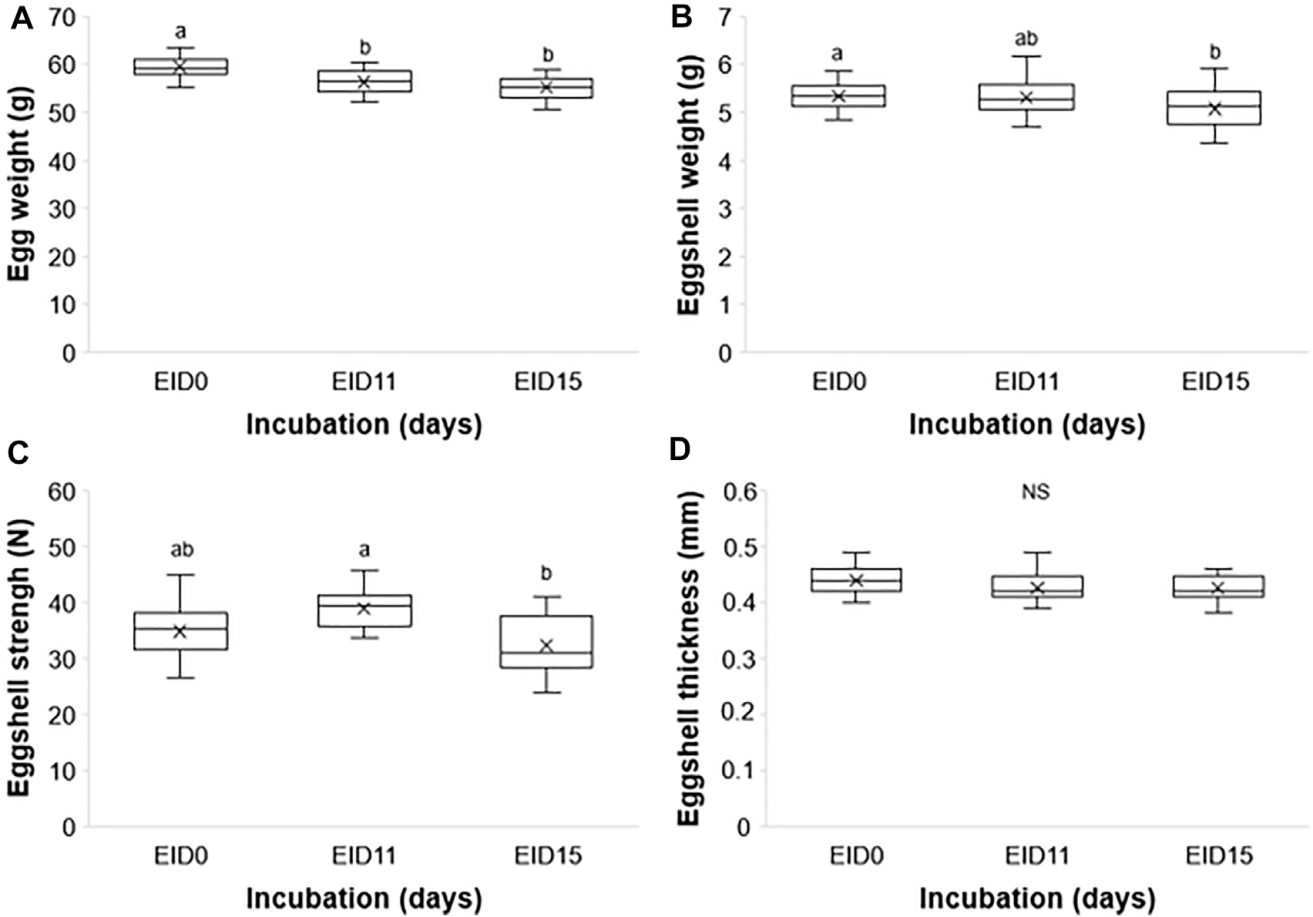
Egg and ES characteristics at EID0, EID11, and EID15. **(A)** Egg weight. **(B)** ES weight. **(C)** ES breaking strength. **(D)** ES thickness. *n* = 30 per developmental stage. Different letters indicate values that are significantly different (*p* < 0.05) between developmental stages EID0, EID11, and EID15.

The egg weight significantly decreases between EID0 and later stages, EID11 and EID15 (59.24 ± 2.29 g at EID0 vs. 56.24 ± 2.55 g and 55.19 ± 2.41 g at EID11 and EID15, respectively; *p* < 0.001; Figure 2A). The ES weight decreases similarly between EID0 and EID15 (5.34 ± 0.31 g at EID0 vs. 5.08 ± 0.41 g at EID15; *p* = 0.037; Figure 2B) but no significant difference was found between EID0 and EID11 nor between EID11 and EID15. In addition, we observed a significant decrease of the ES breaking strength between EID11 and EID15 (*p* = 0.007; Figure 2C), while no significant difference in ES thickness could be found between EID0 and EID11 (*p* = 0.170), and EID0 compared with EID15 (*p* = 0.110) (Figure 2D).

### Microscopic Observations

**FIGURE 3:**
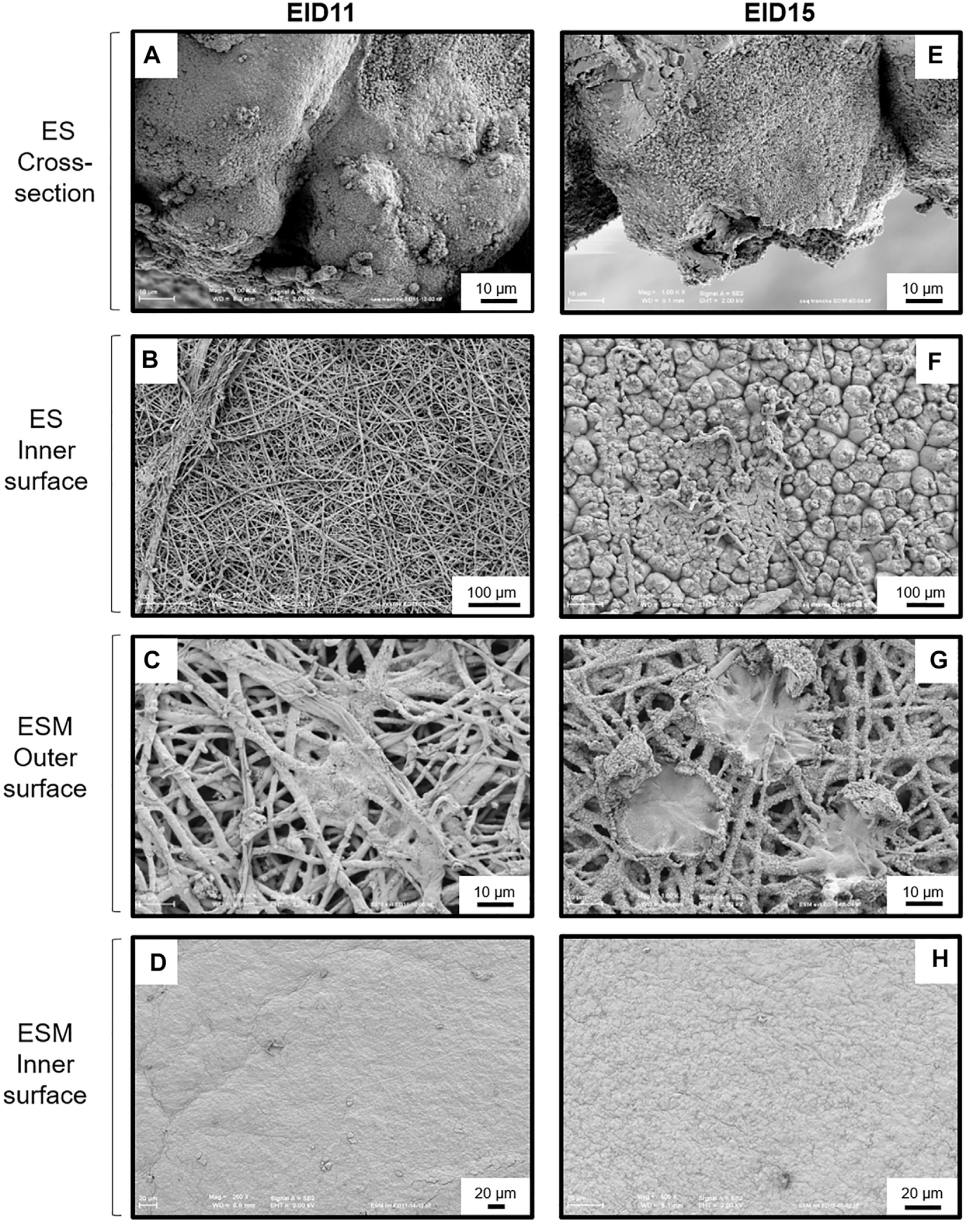
SEM analyses of the structural modifications of the ES and ESM between 11 and 15 days of incubation (EID11 and EID15, respectively). **(A–D)**, left panels, ES and ESM structures at EID11. **(E–H)**, right panels, ES and ESM structures at EID15. **(A,E)** Eggshell (ES) cross-section. **(B,F)** ES inner surface (in contact with ESM outmost sublayer). **(C,G)** ESM outer surface (ESM outer sublayer in contact with ES). **(D,H)** ESM inner surface (ESM inner sublayer in contact with the chorionic epithelium of the CAM). An illustration of samplings is given in Figure 1. Each micrograph is representative of five independent samples (*n* = five eggs).

Analyses of ES cross section between EID11 and EID15 (Figures 3A,E, respectively) illustrate the deterioration of the inner ES at the base of ES cones. The fibers visible on the micrograph corresponding to the ES inner surface at EID11 (Figure 3B) suggest that outer ESM remained tightly attached to the ES after separation while at EID15 (Figure 3F), the inner surface of the ES essentially contains eroded cones, with no visible ESM fibers. ESM outer surface (in contact with inner ES) is characterized by interlaced proteinaceous fibers at EID11 that correspond to inner ESM (Figure 3C), while after 15 days of incubation (EID15, Figure 3G), ESM outer surface exhibits structures that likely correspond to the degraded tips of three ES cones (mammillary knobs), embedded in ESM fibers. At EID15, during the careful separation of the ESM from ES, part of ES cones (mammillary knobs) remained associated with ESM (Figure 3G vs. Figures 3E,F) while the upper part of ES cones detached and broke. In contrast, the ESM inner surface that is in contact with CAM (Figure 1) displays a smooth aspect at EID11 (Figure 3D) and a more granular/cracked aspect at EID15 (Figure 3H); this corresponds to the limiting membrane that lines the inner ESM (Tan et al., 1992).

**FIGURE 4:**
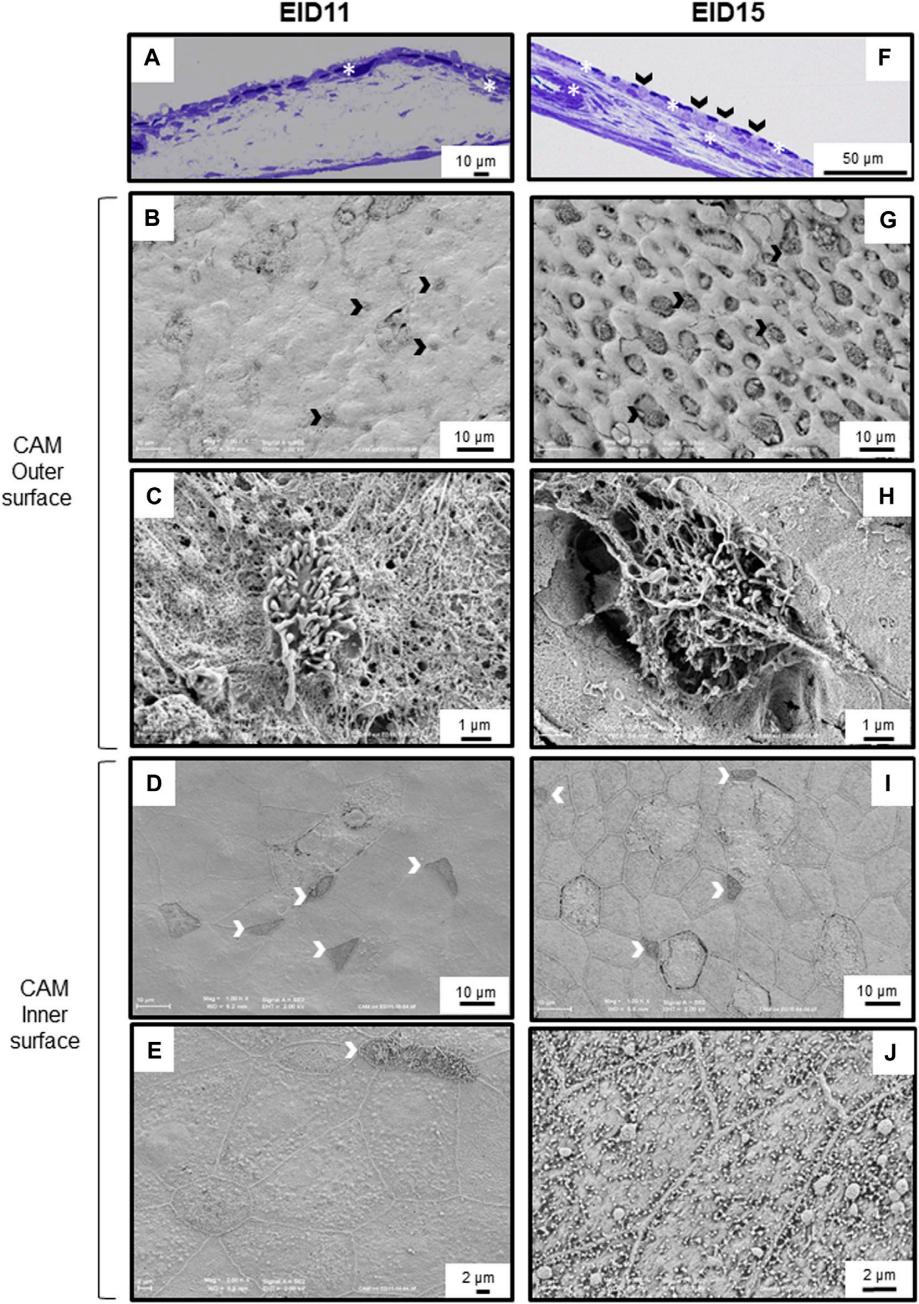
Morphological modification of the CAM between 11 and 15 days of incubation (EID11 and EID15, respectively). **(A–E)**, left panels, CAM morphology at EID11. **(F–J)**, right panels, CAM morphology at EID15. **(A,F)**, Toluidine Blue stained cross-section of the CAM. A large capillary is visible at EID15 [**(F)**, white asterisk]. **(B,G,C,H)**, CAM outer surface (chorionic epithelium in contact with inner ESM). Black arrowheads in **(A,F,B,G)** correspond to some VC cells that characterize the chorionic epithelium. A higher magnification of one of these VC cells is shown in **(C,H)**. **(D,I,E,J)**, CAM inner surface (allantoic epithelium in contact with allantoic fluid). White arrowheads correspond to cells with filopodia. White asterisks indicate capillaries. An illustration of samplings is given in Figure 1. Each micrograph is representative of five independent samples (*n* = five eggs).

Concomitantly, the CAM undergoes major phenotypic changes associated with a progressive cell type differentiation that gradually increases in complexity between EID11 and EID15. The chorionic epithelium contains many capillaries at both EID11 and EID15 (Figures 4A,F, asterisks). All three sublayers of the CAM, namely the chorionic epithelium, the mesoderm and the allantoic epithelium (Figure 1) show significant morphological changes between EID11 and EID15 (Figures 4A,F). At EID15, the mesoderm is characterized by large vessels forming the capillary plexus (Figure 4F, asterisks) that are lacking at EID11 (Figure 4A). It is noteworthy that the CAM exhibits irregular thickness. Some villus-cavity (VC) cells are already detectable at EID11 (Figure 4B, black arrowheads) and their number increased significantly at EID15 of incubation. At higher magnification, the VC cells display villi that project outwards (Figures 4C,D). These villi are thought to penetrate the ESM layer to reach the mammillary knobs. The allantoic epithelium also undergoes morphological modifications. At EID11, some cells start to differentiate but they are still poorly structured (Figure 4D), although at least three distinct cell types can be defined (Figures 4D,E). Some cells contain microvilli (white arrowheads, triangular shape, (type 1 cells), some others have a rounded shape (type 2 cells), while the third group have a pentahedral or hexagonal shape and exhibit a granular aspect (type 3 cells). Type 2 cells are surrounded by 6–7 type 3 cells and form a sort of flower (Figure 4E). These cellular assembly is also visible at EID15 (Figure 4I). At EID15, cells are well structured and differentiated (Figure 4I). At higher magnification, the type 3 cells are seen to possess granules and numerous short microvilli that project towards the allantoic lumen (Figure 4J).

## Discussion

Through evolution, the ES has been shaped to resist physical and pathogen challenges from the external environment, while satisfying the metabolic and nutritional needs of the developing embryo. Indeed, the ES regulates gas and water exchange, and serves as a calcium store (Hincke et al., 2012). The progressive solubilization of ES minerals during incubation to meet the calcium needs of the embryo is paralleled with the loss of ES matter (reduced weight, Figure 2B), the decrease of ES breaking strength (Figure 2C) as previously published (Halgrain et al., 2022), although no significant decrease in ES thickness was found between EID11 and EID15 (Figure 2D). The loss of total egg weight (Figure 2A, about 8% between EID0 and EID15) is mainly due to water loss during incubation, which in turn increases the volume of the air cell. Notably, this water loss is limited by the outermost layer of the eggshell termed the cuticle that forms a pore plug at the surface of the eggshell (Kulshreshtha et al., 2022). An egg loses about 12–14% of its weight until pipping (Meijerhof and Van Beek, 1993; Nakage et al., 2003).

The chicken ES is a complex and well-defined structure. From the inside (in contact with the ESM) to the external surface, the mineralized ES is characterized by the mammillary layer (or mammillae), where biomineralization is initiated, the palisade layer (responsible for most of ES resistance to fracture), the transitional vertical crystal layer and, finally, the cuticle (Simons, 1971; Chien et al., 2009a, b; Solomon, 2010; Hincke et al., 2012). ES resorption during incubation mainly occurs from the calcium reserve bodies located in the mammillary region (Tyler and Simkiss, 1959; Simons, 1971; Bond et al., 1988; Dennis et al., 1996; Chien et al., 2008). This resorption is illustrated by the erosion of the mammillary knobs (Figure 3A/Figure 3C), which leads to the detachment of the ESM fibers (Figure 3E/Figure 3G) (Bellairs and Boyde, 1969; Simons, 1971; Bond et al., 1988; Chien Y.-C. et al., 2009). Collectively, these data revealed that at EID15, the ES innermost region becomes more fragile, which explains the slight decrease in ES breaking strength (Figure 2C). ES resorption also results in loosening the ESM fibers that penetrate the tips of the mammillae (Figures 3E,F vs. Figures 3A,B), the latter remaining tightly attached to ESM fibers after careful manual separation of ES and ESM (Figure 3G). In the last week of incubation, the membrane is not only loose but exhibits a large amount of solid calcium carbonate around each mammillary core (Tyler and Simkiss, 1959). Solubilized calcium from these mineral structures has long been thought to cross the ESM fibrous net to be assimilated by the growing embryo (Plimmer and Lowndes, 1924). It is now well established that the solubilization of ES minerals and their subsequent uptake is mediated by the chorioallantoic membrane (CAM) (Simkiss, 1961; Leeson and Leeson, 1963, Terepka et al., 1969), a vascularized extraembryonic structure that is in contact with the inner surface of ESM and that is physically connected to the embryo. The limiting membrane that covers the inner ESM also undergoes significant morphological changes between EID11 and EID15, starting from a smooth surface to a more granular surface (Figure 3H vs. Figure 3D). During quail embryonic development, ESM was reported to be become thinner, which was proposed to facilitate water exchange and chick hatching (Yoshizaki and Saito, 2002). The thinning of ESM may result from the solubilization of the inner ESM (limiting membrane). The underlying mechanisms of such a morphological change remain unknown but might result from CAM cellular and possibly hydrolytic activities. We hypothesize that such a degradation of ESM limiting membrane may expose the fibers of the inner ESM and could explain the observed change in texture (Figure 3H).

The CAM is an extra-embryonic structure that results from the fusion of the chorionic and allantoic membranes around day 5 of incubation (Freeman and Vince, 1974; Gabrielli et al., 2001). This highly vascularized structure surrounds the embryo, adheres to the acellular inner ESM and covers all other egg structures by 10th-11th day of incubation (Romanoff, 1960) (Figure 1). As previously reported (Ribatti et al., 2006; Maksimov et al., 2006), at EID11, we observed small vessels lining under the chorionic epithelium (Figure 4A) and large capillaries in the mesoderm at EID15 (Figure 4F). The capillary network was reported to cover 87% of the chorionic epithelial surface at EID12 and 89% at EID15 (Maksimov et al., 2006). The presence of these vessels close to the ES minimizes the diffusion distance for the exchange of respiratory gases (carbon dioxide and oxygen) as well as the transport of mobilized calcium ions from the eggshell to the embryo (Ribatti et al., 2006). The apical surface of its innermost epithelium (allantoic epithelium) bathes in the allantoic fluid (Figure 1). The CAM development results in a three–layer structure, each of them having a specific function. The chorionic epithelium that is in close contact with the inner ESM, contains two-functional cell-types involved in the dissolution and the transport of calcium from the ES to the embryo, the villus-cavity (VC) cells and capillary-covering (CC) cells, respectively (Leeson and Leeson, 1963, Coleman and Terepka, 1972a, b; Owczarzak, 1971; Dunn et al., 1981; Gabrielli and Accili, 2010) (Figures 4F–H). The intermediate layer, the mesoderm, is characterized by large capillaries at EID15 (Figure 4F) and is the site of the early development of the extraembryonic vascular system (Gabrielli and Accili, 2010). The allantoic epithelium, the innermost layer, constitutes an important barrier against the acidity of the allantoic fluid and intraluminal toxic contents. It is involved in the water and electrolytes reabsorption from the allantoic fluid. This layer is characterized by the presence of mitochondria-rich cells, allantoic epithelial cells and granule-rich cells (Gabrielli and Accili, 2010; Makanya et al., 2016). During the second half of incubation, CAM rapidly develops and becomes fully mature by EID15-16 (Makanya et al., 2016). This tissue maturity is accompanied by profound cellular differentiation in all three CAM sublayers (Figure 4F vs. Figure 4A) (Makanya et al., 2016). We observed major textural modifications of the surface of the chorionic epithelium between EID11 and EID15 with VC cells progressively extruding outwards (Figures 4B,G, respectively). These cells have been reported to have a major role in proton secretion for solubilization of the ES calcium carbonate (Narbaitz et al., 1995; Gabrielli et al., 2004). Interestingly, similar villi cells are observable at the allantoic epithelium but also two other distinct cell types (Figures 4D,I). It is likely that these cells are somehow involved in acid-base homeostasis and in recycling of ions and metabolites from the allantoic fluid (Gabrielli and Accili, 2010). The full repertoire of molecular components associated with CAM differentiation and transport of minerals from the ES to the embryo remain mostly incomplete, although some candidates have been reported previously (Gabrielli et al., 2004; Matschke et al., 2006; Halgrain et al., 2022).

Altogether, these data give additional evidence for significant structural alterations in the ES that occur during incubation. Although weakening of the ES facilitates the emergence of the hatchling, it may also increase the risk of trans-shell contamination by external pathogens as ES resistance and solidity is weakened. This weakening might also facilitate carbon dioxide and oxygen exchanges *via* CAM capillaries from the chorionic epithelium and the mesoderm (Figure 4F, Ribatti et al., 2006). Interestingly, the degradation at the bases of the ES mammillae between EID11 and EID15 (Figures 3A,E) has only a small impact on ES breaking strength (Figure 2C). We speculate that the CAM structural characteristics provide another layer of egg defense: the CAM may minimize the negative impact of ES weakening during incubation by absorbing physical shocks. Since the CAM provides a physical barrier between the weakened eggshell and the water-rich allantoic fluid, this extra-embryonic structure could also help limit water loss. Aside from its role as a physical barrier, the CAM may also participate in egg defense by its capability to stimulate molecular and cellular responses to microbial stimuli (Hincke et al., 2019; Réhault-Godbert et al., 2021). Deposition of lipopolysaccharide (LPS, an essential component of the outer membrane of Gram-negative bacteria) on the CAM after ES opening was shown to induce a significant inflammatory response that is illustrated by the recruitment of heterophils and monocytes to the immunostimulated site, *via* the CAM vasculature (Valdes et al., 2002). Following viral infection, the CAM expresses interferon (Isaacs and Lindenmann, 1957). However, data related to the role and the response of the CAM after microbial stimulation are relatively scarce. Under physiological conditions, the CAM contains constant levels of some antimicrobial components (ovotransferrin, riboflavin binding protein, lysozyme, ovalbumin-related protein X, TENP and ovoinhibitor, Cordeiro and Hincke, 2016) but microbial stimulation is likely to reveal yet unknown cellular and molecular processes that may be of major interest towards our understanding of innate immunity of fertilized egg.

In addition, we hypothesize that the solubilization of ES mineral by the CAM may also lead to the release of occluded antimicrobial proteins (Hincke et al., 2019). Indeed, the organic matrix of the ES contains a number of antimicrobial proteins, including lysozyme, ovotransferrin, ovocalyxin-32 and -36 (OCX-32 and OCX-36) and ovocleidin-17 (OC-17) (Hincke et al., 2000; Gautron et al., 2001a, b; Gautron et al., 2007; Wellman-Labadie et al., 2008). Similarly, the ESM possesses other proteins associated with innate immunity such as TENP (BPIFB7), AvBD-11 (GAL11), MSMB3, ovalbumin-related protein X (Gautron et al., 2007; Cordeiro et al., 2013; Cordeiro and Hincke, 2016). The presence of most of these proteins in ESM at EID15 and at later stages could reflect their solubilization from the ES and/or their secretion by the CAM.

This study presents for the first time the concomitant morphological changes of the ES, ESM, and the CAM during embryonic incubation, using scanning electron microcopy. It provides new perspectives to understand the interaction between mineralized, organic and cellular structures (ES, ESM and the CAM, respectively). Our data should stimulate further studies to decipher how egg defense, gas exchanges and water loss are regulated during the second half of egg incubation, in response to ES weakening.

## Data Availability

The original contributions presented in the study are included in the article. Further inquiries can be directed to the corresponding author.
